# Perinatal Brain Injury and Inflammation: Lessons from Experimental Murine Models

**DOI:** 10.3390/cells9122640

**Published:** 2020-12-08

**Authors:** Aisling Leavy, Eva M. Jimenez Mateos

**Affiliations:** Discipline of Physiology, Trinity Biomedical Sciences Institute, School of Medicine, D02 R590 Dublin, Ireland; leavyai@tcd.ie

**Keywords:** perinatal brain injury, neonatal encephalopathy, inflammation, hypoxia, hypoxia–ischemia

## Abstract

Perinatal brain injury or neonatal encephalopathy (NE) is a state of disturbed neurological function in neonates, caused by a number of different aetiologies. The most prominent cause of NE is hypoxic ischaemic encephalopathy, which can often induce seizures. NE and neonatal seizures are both associated with poor neurological outcomes, resulting in conditions such as cerebral palsy, epilepsy, autism, schizophrenia and intellectual disability. The current treatment strategies for NE and neonatal seizures have suboptimal success in effectively treating neonates. Therapeutic hypothermia is currently used to treat NE and has been shown to reduce morbidity and has neuroprotective effects. However, its success varies between developed and developing countries, most likely as a result of lack of sufficient resources. The first-line pharmacological treatment for NE is phenobarbital, followed by phenytoin, fosphenytoin and lidocaine as second-line treatments. While these drugs are mostly effective at halting seizure activity, they are associated with long-lasting adverse neurological effects on development. Over the last years, inflammation has been recognized as a trigger of NE and seizures, and evidence has indicated that this inflammation plays a role in the long-term neuronal damage experienced by survivors. Researchers are therefore investigating the possible neuroprotective effects that could be achieved by using anti-inflammatory drugs in the treatment of NE. In this review we will highlight the current knowledge of the inflammatory response after perinatal brain injury and what we can learn from animal models.

## 1. Neonatal Encephalopathy and Neonatal Seizures

Neonatal encephalopathy (NE) is a multi-aetiology condition characterised by disturbed neurological function in the first few days of life for babies past 35 weeks of gestation, and comes with a high risk of morbidity and mortality [1]. NE affects an estimated 3 to 5 in 1000 births, with higher incidences observed in lower income countries [1]. Seizures are a common symptom of NE and a neurological emergency in neonates, strongly associated with mortality and the development of significant neurodevelopmental disabilities [2]. Although no consensus has been reached on the definition of neonatal seizures, they are widely accepted to be transient electrographic changes in the brain resultant of excessive, synchronous or abnormal neurological function, presenting with or without clinical signs and occurring in the first 28 days of life for a full term neonate, or before 44 weeks gestational age for a premature neonate [3]. Due to a variety of factors, such as differences in study methodology, national economic status and inter-observer variation, the incidence of neonatal seizures varies markedly, ranging from 0.95–5.0 in every 1000 births in high income countries [3,4] to incidences as high as 39.5 in 1000 births in low income countries [5]. Mortality from neonatal encephalopathy has decreased in recent decades; however, neurological ramifications are still prevalent [2].

### 1.1. Aetiology

A broad range of conditions are responsible for NE onset, such as Hypoxia-Ischemia Encephalopathy (HIE), which is by far the most common cause, placental abnormalities, maternal factors, perinatal infections, metabolic disorders and coagulopathies; and in a small number of cases the exact aetiology is never determined [1]. As the most common cause of NE, HIE is the aetiology of choice when inducing NE in animal studies, particularly in murine and rat models. It is also the most common cause of neonatal seizures, with over half of infants with HIE developing neonatal seizures [6]. For the purpose of this review, we will focus on the hypoxia–ischemia (first characterized by Rice and Vannucci [7]) and hypoxia-only models, the most common causes of NE and the most widely studied.

#### Classification of Neonatal Seizures in Murine Models of Neonatal Encephalopathy

Two main types of seizures have been observed in the two main murine models of HIE, the hypoxia–ischemia and hypoxia-only models [8,9]. The first type is defined by a burst of high amplitude spikes occurring at regular intervals and returning to baseline between discharges [8]. The second type is characterized by spike-and wave pattern increasing up to 1–2 Hz, and it is mainly observed after reoxygenation or reperfusion [8].

Both types of seizures have been observed in both models, the hypoxia–ischemia and hypoxia-only models; however, they have different characteristics. In the hypoxic ischemic rat model, the durations of the events of the high amplitude spikes (first type) were on average 220 s and they started within 30 min of the ischaemic phase [8]. In contrast, in the mouse hypoxia-only model, the high amplitude spikes lasted for 12.9 s and they started within the 5 min of the hypoxic phase [9]. Importantly, in both models the seizure-like activity was associated with changes in behaviour. During the discharges in the rat model, features such as myoclonic jerks, vocalization, head bobbing, and repetitive clonic jerks were observed [8]. In the hypoxia-only mouse model, the behaviours associated with the discharges included circling, swimming, pedalling, spasms and shaking [9]. However, these differences in behavioural seizures could be more related to differences in species than electrographic characteristics of the seizure. Significantly, we do not know how these types of seizures may contribute to the pathology of neonatal seizures; to date, no correlations have been seen between the burst of high amplitude spikes and spike-wave patterns post-insult and molecular markers or neurological outcomes, and how they can contribute to the re-wiring of the brain. 

The role of inflammation in neonatal seizures is not fully understood. We know that targeting inflammation before hypoxia reduced the number of seizure events and the duration of seizures [10,11]. The mechanism by which this targeting of inflammation reduces seizure activity will require further investigation. Evidence from in vivo experiments supports the role of the cytokine IL-1β signalling in hyper-excitability, as intracerebral injections of IL-1β exacerbate the seizure phenotype of the pro-convulsant agents kainic acid (kainic-acid receptor agonist) and biccuculine (GABA-A receptor antagonist) [12,13]. Furthermore, in transgenic mice, overexpression of IL1-receptor was protective against seizure onset [13]. The underlying mechanism of how IL-1β regulates seizure activity is not fully understood. Evidence from the febrile rat model and adult epilepsy shows that regulation of NMDA receptor activation via IL-1β may be the responsible for the increase seizure activity [12]. Similarly, another pro-inflammatory cytokine, TNFα, increases AMPA-receptor density on the membrane, contributing to the hyper-excitability [14].

In summary, evidence for experimental models shows that anti-inflammatory treatment could reduce seizure activity and cytokines may regulate the activity of AMPA and NMDA receptors, but further studies will be necessary to identify the mechanisms underlying those processes in the immature brain.

### 1.2. Current Treatments for Neonatal Encephalopathy

Babies suffering from neonatal encephalopathy and seizure require urgent treatment to improve their conditions and reduce mortality and morbidity. Currently, the main treatments for neonates with suspected NE are therapeutic hypothermia and phenobarbital (PhB). Therapeutic hypothermia is the only approved non-drug treatment for infants with NE, despite its low response rate. Hypothermia only works in infants with moderate hypoxia and is only effective in reducing the duration of seizures (without reducing the number of ictal events). In the moderate to severe cases (babies suffering from moderate to severe hypoxia), hypothermia is the only therapy option, despite its limited effect, and 50% of children require a second intervention [6]. Importantly, hypothermia does not improve the neurological outcomes after NE [6].

Phenobarbital (PhB), a positive allosteric modulator of GABA-A receptors, is the first line drug for the treatment of neonatal seizures. PhB has a response rate of 50%, and exacerbates the seizure phenotype in the remaining cases [15,16]. This effect of PhB is based on its mechanisms of action and the specific nature of the immature neurons. In immature neurons, activation of the GABA-A receptor results in an increase of intracellular Cl^−^ levels. The high intracellular concentration of Cl^−^ causes neurons to depolarise (activate) rather than hyperpolarise—the typical inhibitory effect of GABA in the mature brain [17].

#### Challenges of Current Drug Treatments in the Clinic

Although PhB has a poor therapeutic profile, it is still recommended as a first-line treatment by the World Health Organisation (WHO), with phenytoin and midazolam as second-line therapies. However, the current anti-convulsant drugs (ACDs) in the clinic present two main risks:

1. Preclinical models have shown that ACDs can induce neurotoxicity and neuronal apoptosis in the immature brain [18,19,20]. With similar results to those observed in a mouse model of hypoxia, PhB itself induces neuronal damage in neonates; and when given as a treatment for hypoxia-induced seizures, it exacerbates the damage [20].
2. Current ACDs affect neurogenesis, synaptogenesis and synaptic plasticity, resulting in unwanted neuropsychiatric outcomes [20,21,22]. Indeed, P7 mice pups receiving PhB presented anxiety-like behaviour and detrimental hippocampal function in adulthood [20]. Furthermore, PhB given as a treatment for neonatal seizures does not improve the lasting anxiety-like behaviour and hippocampal-dependent memory of the hypoxic mice [20]. Supporting the pre-clinical animal model, infants treated with PhB show a strong decrease in cognitive and motor scores at 24 months of age compared to untreated infants [23].

Similarly to current ACDs, poor results were found in the last clinical trial for the treatment of neonatal seizures using the new drug bumetanide (NEMO1; NCT01434225) [24]. Bumetanide, a blocker of the potassium co-transporters (NKCC1 and NKCC2), was observed to reduce seizures in a kainic-acid animal model [18]. Years later, two clinical trials were initiated to establish the safety and efficacy of bumetanide in infants. However, both trials concluded due to the toxic effects of the drug (dehydration, hypotension and permanent hearing loss) and limited evidence of seizure reduction [24]. These studies show the need for understanding the mechanisms underlying neonatal encephalopathy and investigating inflammation as a novel therapeutic target.

## 2. Experimental Animal Models of Hypoxia and Hypoxia–Ischemia

Much research has been conducted to develop different animal models of hypoxia and hypoxia–ischaemia with the aim of finding models which are as reflective as possible of the human condition following neonatal hypoxia. The first model for hypoxia–ischemia in neonates was developed by Rice-Vannucci in 1981 [7]. While this is a very valuable model, it has been associated with high variability in infarct area and symptoms. Modifications of this original model have been developed to reduce variability (Table 1). The most common modifications for this model include levels of oxygen administered during the hypoxia treatment period and the postnatal day at which the treatment is administered, resulting in slightly different phenotypes. Importantly, independent of the modifications to the original model by Rice-Vannucci, all of the hypoxia–ischemia models present with the same set of symptoms, including seizure, memory impairment and hyperactivity.

More recently, a model of hypoxia-only has been developed in mice, to mimic the mild–moderate neonatal encephalopathy more commonly caused by birth asphyxia. Similarly to the hypoxia-ischemia murine models, different levels of oxygen, ages and durations have been used (Table 2). 

Nevertheless, the symptoms observed in the hypoxia–ischemia and the hypoxia-only models are related to seizure, memory impairment and hyperactivity, demonstrating that similar mechanisms are probably activated independently of the original insult. Interestingly, the model outlined by Wang and colleagues [68] (Table 2) noted that treatment with melatonin post-hypoxia improved performance in the Morris water maze regarding learning and memory, which could possibly have been due to the melatonin having an anti-inflammatory effect [68].

In summary, we can conclude that the hypoxia–ischaemia and the hypoxia-only models have similar long-lasting neurological outcomes, including hyperactivity and memory impairment. They also show that the dose of oxygen administered, along with the duration of the treatment, play integral roles in the behavioural characteristics seen post-hypoxia.

## 3. Inflammation in Neonatal Encephalopathy

Neuroinflammation caused by hypoxia or hypoxic ischemia during the perinatal period contributes to increased risk for neurological deficits and long-term disabilities in children [69]. Inflammation induced by injury results in activation of the resident and peripheral immune cells and production of cytokines. In recent years, inflammation has been implicated in neonatal brain damage following perinatal stress. Induction and activation of microglia and astrocytes are hallmarks of neuroinflammation, which occurs in response to hypoxia and hypoxic ischemia in neonates [11,70]. Studies have shown that exposure of the neonatal brain to hypoxia, thereby causing an inflammatory response, is associated with long-lasting changes to neuronal morphology within the hippocampus and other vulnerable structures of the brain [71]. Circulating cytokines and neuroimmune cells such as microglia are the targets of several studies that are investigating the potential use of these components as biomarkers for neonatal brain injury. Additionally, targeting inflammation improves acute and long-lasting effects after hypoxia and hypoxic ischemia in mice [11,70].

Studies into the inflammatory response post-hypoxia have revealed that HIE is a sexually dimorphic disease, with male infants being far more vulnerable to ischaemic insults. Male infants are also significantly more at risk of suffering from long-term cognitive deficits when compared to female infants with comparable brain damage [72]. In fact, the microglial anti-inflammatory response was more robust in females than in males [72]. More infiltration of peripheral lymphocytes, along with upregulated TNFα and IL10, were observed in males when compared with females. It was also noted that neurogenesis was more highly induced in female HIE brains versus male HIE brains. The conclusions drawn from this study were that the pro- and anti-inflammatory responses are indeed dichotomous with respect to sex, which is integral to the sex-specific chronic HIE outcomes, and that increased induction of neurogenesis in females also contributes to this sex-specific difference [72].

It has been long established that infants born prematurely are more vulnerable to the effects of asphyxia and developing adverse sequelae. Acute asphyxia at birth followed by HIE is more frequently seen in infants born prematurely than infants born at term, and is highly associated with the onset of adverse neurological outcomes [73]. Several studies have found that while HIE still only occurs in a minority of preterm births, it is a significant contributor to severe disability [74,75,76]. Mild HIE in preterm infants can result in white matter injury, even in the absence of abnormalities in neurological exams at discharge [75]. The pattern of injury onset can be more prolonged in preterm infants; in some patients who develop cerebral palsy, there are no white matter lesions; however, long-term studies showed that it may be delayed onset demyelination [77]. As a result of this white matter injury, even in mild cases, the resultant adverse outcomes can be attributed to impaired global and regional connectivity between cortical and subcortical grey matter structures [78]. Evidence has also shown a correlation between preterm births and stunted cortical plasticity in adolescents [79]. Extensive evidence from preclinical studies has strongly implicated CNS and peripheral immune responses in the pathogenesis of HIE and preterm brain injury [69]. Various clinical and human post-mortem studies have shown that chronically upregulated systemic and CNS cytokines and gliosis show strong associations with adverse neurological outcomes [80,81]. Evidence has shown that systemic upregulation of TNFα and IL1β in premature infants is associated with impaired neural functions in the first 72 h of life, followed by cognitive impairment at 2 to 3 years of age [81].

It is clear from both human and animal studies that inflammation and the immune response are key to many aspects of the pathogenesis and pathophysiology of HIE and neonatal brain damage. However, further studies will be necessary to elucidate the underlying mechanisms associated with neonatal brain damage.

### 3.1. Pathogen-Associated Molecular Patterns (PAMPS) and Damage-Associated Molecular Patterns (DAMPS)

**Figure 1:**
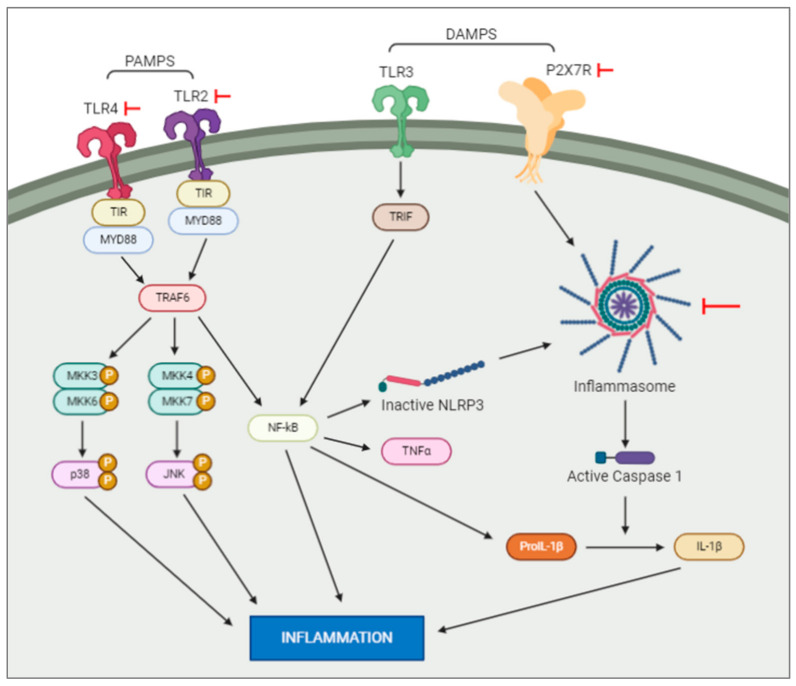
Intracellular pathways activated by DAMPS and PAMPS. DAMPS and PAMPS will bind to the membrane receptors, e.g., TLR and P2X7. This activation will trigger a series of intracellular events resulting in an increase of inflammation. Note: the red arrows represent experiments which have blocked the actions of the elements they are pointed at.

Pathogen-associated molecular patterns, or PAMPs, are normally conserved microbial products such as lipopolysaccharides which activate pattern recognition receptors (PRRs. such as Toll-like receptors (TLRs)) after a bacterial or viral infection. PRR signalling pathways have been shown to initiate cascades that lead to immune cells recruitment to the site of an infection [82]. In contrast to this, danger-associated molecular patterns, or DAMPs, are molecular patterns associated with sterile inflammation, or inflammation instigated without introduction of a pathogenic microbe, as seen after neuronal necrosis. These molecular patterns are released in response to tissue damage, with the same innate pattern recognition systems used in the detection of microbes initiating this sterile inflammatory response [82]. This inflammatory response, followed by tissue repair, is dependent on microglia migration to and from the site of injury [83]. Studies show that DAMPs and PAMPs induce distinctly different inflammatory responses in the neonatal brain (Figure 1). Lalancette-Hebert and colleagues [84] studied the difference between these responses with respect to toll-like receptor 2 (TLR2) expression. It was found that a neonatal mouse model of infection induced TLR2 expression and secretion of inflammatory mediators. Contrasting results were seen in the two neonatal mouse models of sterile inflammation (IL-1β injection and MCAO), which showed decreased induction of TLR2 and reduced production of inflammatory cytokines [84]. This study highlights the existence of scenario-specific innate immune responses, depending on the presence of either infections or sterile inflammation, and the necessity of looking for specific therapeutic strategies depending on the original insult. In the following sections we will examine the activation of the most studied family of receptors in hypoxia and hypoxia–ischemia murine animal models.

### 3.2. Toll-Like Receptors in Neonatal Encephalopathy

Upregulation of pattern-recognition receptors, such as TLRs, has been shown after perinatal brain injury. The Toll like receptor (TLR) family consists of nine subtypes (TLR1–9). They recognize a variety of pathogen-associated molecular patterns (PAMPS), including LPS, bacterial DNA and double stranded RNA. The role of TLR in perinatal brain injury has been extensively studied. TLR1, 2 and 7 are up-regulated 24 h after hypoxic ischemia in pups. TLR5 is downregulated and TLR3, 4, 6, 8 and 9 do not change expression. Interestingly, when KO mice for TLR 1 and 2 were subjected to hypoxic ischemia, TLR2 KO improved the infarct volume after hypoxic ischemia, but TLR1 KO did not have an effect. This data showed that TLR2 plays a role in initiating inflammation after perinatal brain injury [85,86]. Supporting this data, the use of Candesartan Cilexetil, a drug which reduces levels of TLR2, has been shown to improve neuronal damage after hypoxia in mice, and also improve the long-lasting neurological outcomes [11]. Importantly, the response of TLR2 to sterile (e.g., HI) or non-sterile inflammation (e.g., LPS to mimic bacterial infection) differs on the pre-clinical model [84]. Suggesting that, deeper knowledge of the pathways underlying TLR2 is important for developing new pharmacological treatments.

TLR4, another receptor implicated in DAMP and PAMP functions, has also been studied, due to observations that its inhibition has neuroprotective effects in neonatal brain damage [87]. TLR4 inhibition shortly after injury reduced activation of hippocampal glial cells improved hippocampal neuronal loss later in life and resulted in less severe long-term neurological outcomes [87]. These studies, by targeting receptors which mediate the effects of DAMPs and PAMPs, show the important roles played by these molecules in the mediation of the neuroinflammatory response following hypoxic ischaemic brain injury. Further studies are needed to fully understand these interactions in the neonatal brain post-injury.

### 3.3. Purinergic Signalling Activation after Neonatal Encephalopathy

Extracellular adenosine triphosphate (ATP) is a typical DAMP which acts as a glio- and neurotransmitter in the CNS to modulate functions such as brain excitability and neuroinflammation [88]. It is considered to be a co-transmitter in most neurons of the central and peripheral nervous system, and is released from astrocytes and neurons to act as either a co-transmitter or a sole transmitter [89]. The P2X class of ionotropic receptors, made up of seven distinct receptors, mediates the rapid effects of extracellular ATP by gating sodium and calcium entry into cells [90]. The P2X7 receptor (P2X7R) modulates cytokine production, glial activity and neurotransmitter release following brain injury [90]. P2X7R activation is seen in instances of pathologically high extracellular ATP levels, the likes of which are seen during seizures and brain injury. Downstream signalling of the P2X7R results in microglia activation and the release of interleukin 1β (IL-1β), which is a pro-convulsive inflammatory cytokine [91,92,93]. Evidence has shown that P2X7R is expressed by neurons and acts as a modulator of neurotransmitter release [94,95]. Similarly, each member of the P2Y class of eight purinergic metabotropic receptors is stimulated by ATP, and they are generally associated with slower presynaptic functions, and mediation of trophic signalling in cell differentiation, proliferation and death during development [89]. During epileptic seizures, large quantities of nucleotides enter the extracellular space from neurons and glia due to metabolic limitations [96]. These activate the P2X and P2Y receptors, including P2X7 and P2Y1, which are expressed on both embryonic and adult neural progenitor cells (NPCs). These two receptors regulate NPC functions, causing necrosis and apoptosis, and proliferation, differentiation and migration [97,98]. In a study by Rozmer and colleagues [99], patch-clamp recordings were carried out on hippocampal brain slices from neonate and adult transgenic nestin reporter mice which underwent pilocarpine-induced status epilepticus. This study detected the presence of P2X7R in NPCs in the subgranular zone of the dentate gyrus. Upon activation of these receptors, inward current was recorded near the resting membrane potential of the NPCs. P2Y1 receptor activation, on the other hand, initiated outward current close to the reverse potential of the P2X7R current [99]. It was also noted that the sensitivity of these two receptors was invariably increased. In this model, status epilepticus was preceded by a latency of 5 days after treatment with pilocarpine, and recurrent epileptic fits occurred during this period. Blockade of central P2X7Rs increased the number of seizures experienced, along with their severity. Rozmer and colleagues [99] hypothesised from these results that P2Y1 receptors increase proliferation and migration of NPCs, while P2X7R mediated necrosis and apoptosis may counter these effects, which would otherwise result in chronic recurrent epileptic seizures.

Experiments have been carried out to block the P2X7R in order to fully understand the role of this receptor in perinatal stress and subsequent brain injury. P2X7R is over-expressed in a neonatal mouse model of global hypoxia, and targeting of P2X7R with A-438079, a receptor antagonist of P2X7R, can reduce the number of post-hypoxia neonatal seizures [10]. These results corroborated an earlier study by Mesuret and colleagues [100], which used the same inhibitor to investigate the effects of P2X7R antagonism on early-life seizures in rats. This study also found that P2X7R blockade by A-438079 improved neonatal seizures, and suggested A-438079 could be used as a treatment for neonatal seizures or paediatric status epilepticus [100]. Similarly, Brilliant Blue G (BBG), a P2X7R-specific inhibitor, inhibits LPS-induced IL-1β release in mouse models of intrauterine inflammation [101], resulting in perinatal brain injury. P2X7R blockade resulted in reduced preterm birth rates, dendritic arborisation and density of cortical neurons, and improved performance for offspring in neuromotor tests [101]. These results supported the role of IL-1β as a key mediator of perinatal brain injury. Further studies corroborated the neuroprotective effects of P2X7R blockade, with da Silva and colleagues [102] showing that in a neonatal rat model of LPS-induced inflammation, pharmacologic blockade of P2X7R in the neonatal period using BBG has neuroprotective effects that persist into adulthood [102].

### 3.4. Cytokines and Chemokines in Neonatal Encephalopathy

Cytokines and chemokines, such as tumour necrosis factor α (TNFα) and interleukin 1β (IL1β), are released by microglia and astrocytes in response to hypoxic ischaemic injury, amplifying inflammatory cascades that recruit monocytes and neutrophils to the site of injury [103]. Studies have demonstrated an association between more adverse outcomes following perinatal brain damage and pro-inflammatory cytokines, such as TNFα, IL1β and IL6 [104]. These cytokines are released by astrocytes, neurons and microglia and are associated with HIE. A study by Liu and colleagues [104] demonstrated this by studying the peripheral blood levels of TNFα and IL1β of human neonates with HIE and control neonates. It was found that neonates with HIE consistently had higher levels of TNFα and IL1β, and there was a positive correlation between IL1β levels and HIE severity [104]. Chemokines also play a pivotal role in the inflammatory response following NE or HIE, due to their roles in inflammatory cell trafficking and leukocyte activation [105]. When HIE is modelled in rats [106], upregulation of alpha-chemokines such as macrophage inflammatory protein 2 (MIP2) and beta-chemokines such as MIP1α, MIP1β and CCL5 is induced, followed by the expression of lymphocyte markers in the site of infarction. This inflammatory response persisted beyond the neonatal period in this rat model, indicating that this acute inflammation may trigger a chronic inflammatory response [106].

#### 3.4.1. Interleukin 1β, IL1β

There is no doubt that IL-1β levels are increased after neonatal brain injury. Hypoxic ischemia and hypoxia-only both cause acutely increased IL1β levels after the original insult [10,11]. However, it is not clear whether this increase of IL1β is sustained over time. Results from the hypoxia–ischemia model showed increases in IL1β 6 days and 14 days after reperfusion. However, in the hypoxia-only model, IL1 β levels returned to normal levels after 72 h [11].

Supporting the role of IL1 β in ischemia, administration of type 1 interleukin receptor (IL1R1) antagonist or blocking antibodies ameliorates damage induced by excitotoxicity and/or ischemia. IL1β knockdown by lentivirus in vivo can also improve the damage caused by neonatal HI [10]. Further analysis will be necessary if these results can be reproduced on the hypoxia-only model where the induction of IL1β is transient.

#### 3.4.2. Interleukin-6, IL6

IL6 has been shown to have a dual function, having beneficial and/or detrimental effects depending on the context. In adult rodents, IL6 has an inflammatory effect in the acute phase, but during the chronic phase, IL6 acts as a neurotrophic factor [70].

In the neonatal brain, IL6 is increased transiently in the first hours after ischemia. Blockers of IL6 have a neuroprotective effect after the ischemic insult. However, no differences were observed between the ipsilateral (hypoxic-ischemic side) and the contralateral side (hypoxic only side), making it difficult to determine the role of IL6 in the neonatal brain. Further studies will be necessary to fully elucidate the role of IL6 during neonatal brain injury.

#### 3.4.3. Tumour Necrosis Factor, TNFα

It is clear that TNFα is increased after neonatal brain injury; however, its role is not clear. TNFα binds to TNF-R1 and TNF-R2. Activation of TNF-R1 activates a caspase signalling pathway, resulting in cell death. In contrast, activation of TNF-R2 induces cell proliferation via the survival Akt signalling pathway. In neonatal hypoxic ischemia in rats, an increase of TNF-R1 has been observed in oligodendrocytes, suggesting that TNFα may play a role in the apoptosis and delayed myelination observed in neonatal brain injury. The role of TNFα will require further analysis to clarify its beneficial or detrimental effect [107].

## 4. Contributions of Central and Peripheral Cells to Neonatal Encephalopathy

**Figure 2:**
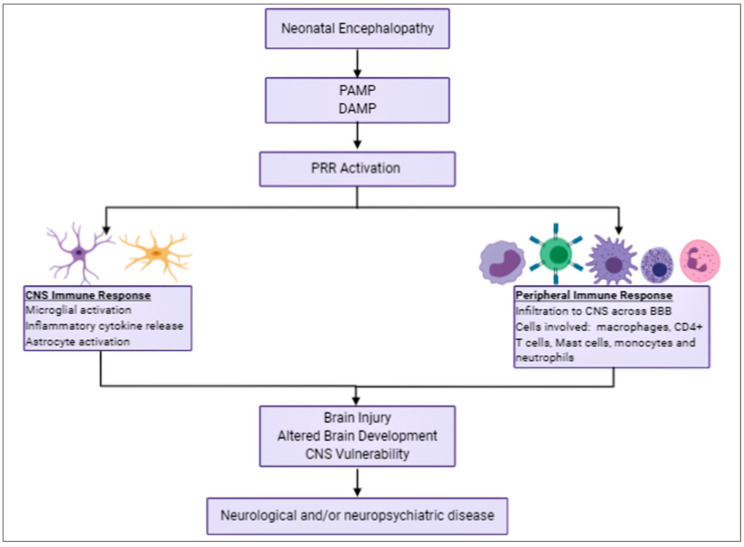
Summary of the inflammatory processes that occur following neonatal encephalopathy (NE), highlighting the cell types involved from both the CNS and peripheral immune response along with the ramifications of NE that persist long after this inflammatory response abates.

Both central and peripheral immune cells contribute to the damage induce after hypoxia-ischemia and hypoxia. In this section, we will discuss how the different cells types contribute to the damage after NE (Figure 2).

### 4.1. Microglia

Microglia are dynamic cells that maintain neurons and neural circuits, and are responsible for mediating the immune response within the brain once activated. When microglia are activated following injury, their roles include cytokine and chemokine release, phagocytosis and antigen presentation. Microglia undergo morphological transformation into their activated state following HIE. Microglial aggregation and activation have been established as pathological markers for HIE, as activated microglia are believed to contribute to HIE and excitotoxic injury [70]. Winerdal and colleagues [107] investigated long-term local and systemic inflammation in a mouse model of HIE. The study showed that in the months following the initial HIE event, the mice showed elevated activation of local and systemic inflammatory response, and suggested that this prolonged inflammation also contributes to potential brain damage following HIE [107]. Another study by Serdar and colleagues [83] investigated microglial phenotypes in the early stages of hypoxic-ischaemic brain injury using a rat model of inflammation-sensitised HI brain injury. This study identified microglia as key mediators of the inflammatory response following injury, with a predominantly pro-inflammatory phenotype adopted in the 24 h post-hypoxia [83]. Studies of this nature open doors for new treatment options to be explored which target microglial processes and morphology to prevent the injurious inflammatory response post-hypoxia.

### 4.2. Astrocytes

Astrocytes are the most abundant of the glial cells in the mammalian brain, and provide many functions within the CNS, including regulation of the extracellular environment, removing excess neurotransmitters and provision of metabolic and structural support for neurons [108,109]. Excessive glutamate release occurs following HIE, leading to overstimulation of the glutamate receptor N-methyl-D-aspartate (NDMA). This overstimulation triggers the excito-oxidative injury cascade by causing excessive Ca2+ influx to the cytosol, resulting in apoptotic cell death in neurons [110]. Contributing to the pathology after hypoxic ischemia, astrocytes have a diminished capacity for glutamate uptake caused by mitochondrial failures [111]. Interestingly, mitochondrial failure was found to be a sexually dimorphic; male pups have a stronger acute response than females, and female pups have a longer reduction in function than males [111].

Wang and colleagues [112] investigated the role of TRVP1 translocation in astrocytic membranes at the onset of HIE-induced epilepsy, potentially identifying this receptor channel protein as a therapeutic target. TRVP1 is a member of the vanniloid transient receptor potential (TRVP) channel family, and is a Ca^2+^ permeable channel, previously studied for its role as a pain receptor in sensory neurons [113]. Using a mouse model, this study ascertained the importance of TRVP1 in the promotion of astrocyte migration, which in turn encourages the infiltration of pro-inflammatory cytokines to the vicinity of neurons and promotes the onset of epilepsy. The results of this study rationalise the potential of TRVP1 as an anti-epileptogenic therapeutic target after HIE [112].

Evidence implicates astrocytes at the onset of damage post-HIE; however, astrocytes and their responses to certain cytokines are also associated with having certain neuroprotective effects. IL-10 released from astrocytes suppressed neuronal apoptosis in response to HIE via the TLR2/NFκB pathway in a rat model of hypoxia-ischemia [114]. Similarly, the astrocyte-derived IL-33 following HIE is upregulated in the first 24 h post-HIE, and the ST2 receptor, the receptor for IL-33, was shown to be upregulated in astrocytes post-HIE [115]. Importantly, exogenous delivery of IL-33 via intraperitoneal injection alleviated the resultant brain injury 7 days post-HIE. Conversely, deficiency of the ST2 receptor exacerbated brain damage and neurological sequelae post-HIE. When mice were treated with IL-33 post-HIE, astrocyte apoptosis was attenuated, and astrocyte response was improved through the ST2 pathway, including released neurotrophic factors essential for neuronal survival against oxygen and glucose deprivation.

The role of IL-6 is HIE is not clear (see section above, “Interleukin-6, IL6”); however, IL-6 has been found to be neuroprotective after HIE due to its effects on astrocytes. Overexpression of IL-6 via mesenchymal stem cell transplantation alleviates neurological sequelae following HIE. The mechanism of this neuroprotective action is not fully understood; however, it has been observed that IL-6 suppresses apoptosis of injured astrocytes [116] and reduces proliferation of reactive astrocytes induced by HIE [117].

### 4.3. Oligodendrocytes

Neurons and oligodendrocytes during development have high metabolic rates, making them more vulnerable to anoxia. During development, grey and white matter injuries have been associated with sub-acute and chronic hypoxia-ischemia. Importantly, white matter injury is common in pre-term babies; however, results are contrasting in term babies, in whom white matter injury has been linked with chronic hypoxia, but not in areas of high metabolic activity like the thalamus [118]. These differences in white matter injury in pre-term and term babies may be a reflection on the maturation of oligodendrocytes in pre-term and full-term babies [69].

The degree of damage on premature neonates correlates with a predominance of late oligodendrocytes progenitor cells (OPCs) in the immature brain. The observed hypomyelination may be due to the death of OPC, stopping oligodendrocyte maturation, and depletion of the number of OPCs [119,120].

It is not clear what the mechanism underlying the damaging of OPCs is in premature babies; evidence has shown that OPCs express the calcium-permeable AMPA receptor. Glutamate activates the AMPA receptor, inducing an increase of intracellular Ca^2+^, resulting in Bax activation, translocation of Bax to the mitochondria and activation of caspase-3 and cell death [121].

Supporting the role of inflammation in myelinations, intraperitoneal injections of IL-1β in the first days of life results in an increase in unmyelinated fibres. Additionally, IL-6 could also induce cell cycle withdrawal and maturation of OPCs [122]. In fact, transgenic mice which overexpressed IL-6 had severe neurological symptoms, including ataxia, tremor and seizures; however, white matter damage was not demonstrated [123].

### 4.4. Peripheral Immune Cells

Peripheral immune cells can cross the blood–brain barrier (BBB) via a number of BBB portals, namely, the parenchymal blood vessels, the meningeal vessels and the choroid plexus [124]. In neonates, low levels of monocytes, macrophages and CD4^+^ T-cells exist in the CSF of healthy neonates for what is believed to be immune surveillance purposes [125]. In response to insults such as HIE, the immature CNS, as seen in neonates, upregulates a number of chemoattractant molecules in response to HIE, including CCL2, CCL3 and CCL7, resulting in recruitment of monocytes from the bone marrow, and CXCL1, responsible for recruiting monocytes to inflamed tissues [126,127]. These guide peripheral immune cells towards the CNS, and as a result, a number of peripheral immune cells infiltrate the CNS and accumulate within.

Accumulations of neutrophils in the brain’s blood vessels following HIE have been observed in numerous clinical studies and experimental models; however, they tend to remain largely contained within the blood vessels [106,128]. It has been hypothesised that neutrophils are implicated in an early-phase temporary impairment of red blood cell and oxygen flow [124].

Studies of mast cells in neonatal HIE have found that these cells excessively express transforming growth factor beta (TGF-β), which encourages the onset of excitotoxic brain injury [129]. Mast cell numbers were also seen to increase in the acute HIE response, with rapid degranulation and release of TNF-α also being observed. Furthermore, inhibition of mast cell degranulation and activation had neuroprotective effects [130,131].

The role of monocytes has been extensively studied in HIE in preclinical models [132]. Significant numbers of peripherally derived MDMs infiltrated and accumulated in the immature CNS post-HIE, with the majority of this invasion occurring one day post-HIE. They also ascertained that inhibition of this accumulation of myeloid cells was neuroprotective in male but not female neonatal mice. This study demonstrated the infiltration of peripheral monocytes, while also supporting the sexually dimorphic nature of the inflammatory response post-HIE.

T cells generally do not cross the BBB; however, higher CD4^+^ T cell numbers is a characteristic of the post-HIE immune response [106]. Maximal CD4^+^ T cell numbers were observed 7 days post-HIE, and these cells persisted within the CNS up to 35 days post-HIE. This recruitment of CD4+ T cells attract the recruitment of CD8+ T cells in a rat model of HIE. Importantly, these cells persisted in the CNS 3 months post-HIE [107]. Supporting this data, Albertsson and colleagues [133] demonstrated in a mouse model that this CD4+ influx was biphasic, with this influx occurring 1 day and 7 days post-HIE.

Immune cell infiltration has been shown to have an important role in neonatal brain injury, with elements from both the innate and adaptive immune systems recruited to the CNS following neonatal HIE. This also shows the complexity of the inflammatory response that is triggered in both the acute and chronic responses to HIE.

## 5. Conclusions

The merits of using anti-inflammatory drugs as treatments for neonatal encephalopathy have been extensively studied. Mounting evidence has shown that the inflammatory components within the brain, such as microglial activation and cytokine signalling, play pivotal roles in the pathophysiology of HIE and neonatal seizures [11,71]. Several studies have investigated the effects of anti-inflammatory drugs as treatments for HIE and neonatal seizures, particularly small molecule drugs, as these are more likely to successfully cross the blood-brain barrier. Thus far, these studies have shown the beneficial neuroprotective effects of these anti-inflammatory molecules in animal models [10,11]. However, several questions remain unanswered regarding the nature of the main inflammatory response induced by perinatal stress. Research so far has been unable to ascertain whether the activated immune cells seen in the post-hypoxia response are central immune cells or are recruited from peripheral tissues. Additionally, research into the effects of anti-inflammatory therapeutics has yet to determine whether the long-term outcomes resulting from neonatal brain damage can be improved by targeting inflammation weeks after the original insult. Various inflammatory pathways have been discussed in this review, particularly the TLR and purinergic pathways which are dictated by DAMPS and PAMPS activation. Those elements which have not been blocked or antagonised by experiments are possible targets for future studies, so that we might fully understand their roles in the post-hypoxia ischemia response.

## Figures and Tables

**Table 1:** Rat and mouse models for hypoxia–ischaemia. The experimental designs employed, along with the resultant behavioural characteristics and O_2_ levels.

O^2^ Levels	Age	Duration	Common Reported Behaviour	References
*Rat*				
0%	P0–P11	5–30 min	Hyperactivity in open field, impaired memory, increase anxiety	[25,26,27,28,29,30,31,32,33,34,35,36,37,38,39,40,41,42,43,44]
2.5–5%	P0–P10	15–30 min	Seizures during hypoxia, worse water maze performance	[44,45,46,47,48,49,50,51,52]
5–8%	P7–P10	15 min–3 h	Increase susceptibility to chemical induced seizures at adulthood	[53,54,55,56,57,58,59,60,61,62]
10%	P7–P9	30 min–6 h	Hyperactivity in novel object task	[63,64,65]
*Mouse*				
0%	P0–P15	20–25 min	Hyperactivity and seizures	[66,67]
5%	P1–P7	15 min–2 h	Seizures, impaired learning	[9,68]

**Table 2:** Experimental design for the hypoxia-only mouse models, along with the resultant behavioural phenotypes.

Species	O^2^ Partial Pressure	Age (Postnatal Day)	T (°C)	Duration of Hypoxia	Reported Behavioural Phenotype	References
C57 Mouse	0%	P3–15	RT	20 min	Electrographic seizures without clinical manifestations.	[67]
Mouse	0%	P0	33, 37, 39	25 min	Both open-field stress-induced and spontaneous motor activity reduced. Hyperactive in the plus maze test. Behavioural disturbances were prevented by the body temperature of 33 °C.	[66]
C57 Mouse	5%	P7	34	15 min	Seizures in pups, reduced curiosity in novel object test, weight loss.	[9]
Mouse	5%	P1	-	2 h	Melatonin improved learning and memory in the Morris water maze.	[68]
